# Tri­methyl­pyrazole: a simple heterocycle reflecting Kitaigorodskii’s packing principle

**DOI:** 10.1107/S205698902200860X

**Published:** 2022-09-02

**Authors:** Steven van Terwingen, Ulli Englert

**Affiliations:** a Institut für Anorganische Chemie, RWTH Aachen University, Landoltweg 1, 52074 Aachen, Germany; Universidad de Los Andes, Venezuela

**Keywords:** close-packing principle, mirror plane, layer structure, C—H⋯N contact, crystal structure

## Abstract

The five-membered heterocycle 1,3,5-trimethyl-1*H*-pyrazole, C_6_H_10_N_2_ (**1**) crystallizes in space group *Pnma* with all non-hydrogen atoms of the mol­ecule on the crystallographic mirror plane. This arrangement has been recognized as favorable with respect to space filling by Kitaigorodskii and Wilson, pioneers in the field of crystal packing; *Pnma* represents a particularly rare space group for residues exclusively in a general position. Neighboring mol­ecules in **1** inter­act *via* non-classical C—H⋯N bonds in the plane and C—H⋯π contacts between adjacent layers. In *Pnma*, crystallographic inversion relates dipolar mol­ecules located on successive mirror planes and results in their head-to-tail arrangement. The inter­layer distance in the [010] direction is closely related to the van der Waals radii of C and N.

## Abstract 2

The title compound represents a textbook example for the arrangement of small mol­ecules and space-group symmetry: in space group *Pnma* the mirror planes should be occupied to ensure efficient space filling.

## Introduction

Aleksander Kitaigorodskii was already working on his principle of close packing in the 1940s, at a time when structure analysis *via* single-crystal diffraction was still not fast and routine. We recall that about 20 years later, in 1965, the archives of the Cambridge Crystallographic Data Centre comprised only 3000 structures. Kitaigorodskii’s finding that void space in crystals is in general unfavorable enabled him to rank certain space groups as more or less suitable for close packing. It took considerable time before Kitaigorodskii’s ideas were appreciated in the western world (Kitaigorodskii, 1961 ▸, 1965 ▸, 1973 ▸). The term *symmorphic* refers to space groups that exhibit a special position with the same symmetry as the crystal class (Chapuis *et al.*, 2022 ▸). A. J. C. Wilson expanded these original ideas (Wilson, 1993*a* ▸) and coined the term *anti­morphic* space groups (Wilson, 1993*b* ▸), which only possess symmetry elements associated with a favorable packing, *i.e*. screw axes, glide planes and inversion centers. In contrast to Kitaigorodskii, W. Nowacki explained the statistical preference for certain space groups by their ability to form a favorable dipole arrangement rather than an efficient packing (Nowacki, 1943 ▸, 1951 ▸). An excellent summary of the close-packing principle and its consequences for space-group frequencies, together with other packing criteria, was published by Brock & Dunitz (1994 ▸). 
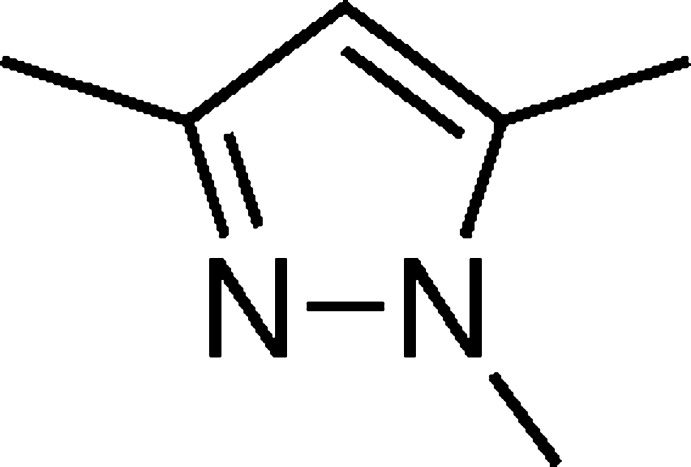


In this contribution, we present the crystal structure of the simple heterocycle 1,3,5-trimethyl-1*H*-pyrazole (**1**) in space group *Pnma* and describe its crystal packing in the context of Kitaigorodskii’s and Wilson’s ideas.

## Results and Discussion

**Figure 1:**
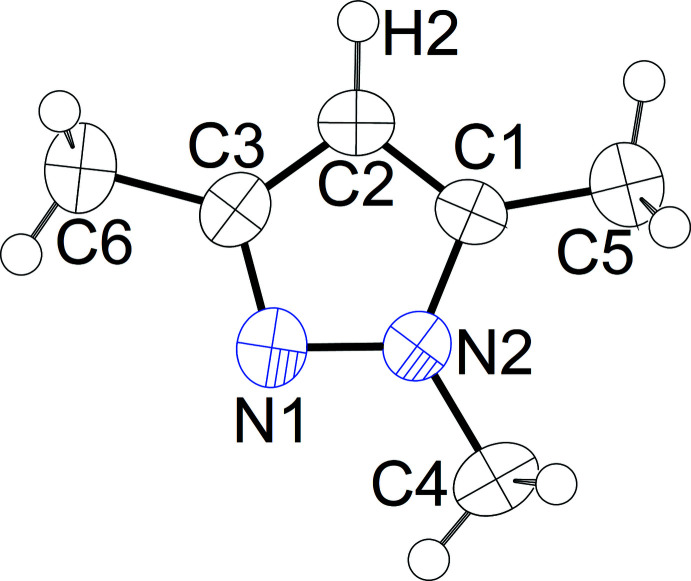
Displacement ellipsoid plot (Spek, 2020 ▸) of a mol­ecule in **1**; ellipsoids are drawn at 70% probability, H atoms are shown as spheres of arbitrary radii. Selected distances (Å) and angles (°): N1—N2 1.358 (4), N2—C1 1.351 (4), C1—C2 1.360 (5), C2—C3 1.392 (4), N2—C4 1.448 (4), C3—N1—N2 104.2 (3), C1—C2—C3 106.6 (3).

All non-hydrogen atoms in **1** occupy a crystallographic mirror plane in space group *Pnma* (Wyckoff position 4*c*), resulting in a strictly planar scaffold. A displacement ellipsoid plot of a heterocyclic mol­ecule is shown in Fig. 1 ▸.

Compared to other simple pyrazoles, this is a unique property as most of them do not crystallize in space groups exhibiting a mirror plane, *e.g.* 1*H*-pyrazole (space groups *Pna*2_1_ and *Pbcn*; Sikora & Katrusiak, 2013 ▸), 3,5-dimethyl-1*H*-pyrazole (space group *R* 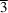 *c*; Baldy *et al.*, 1985 ▸) or 1,5-dimethyl-1*H*-pyrazole-3-carb­oxy­lic acid ethyl ester (*P* 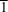 ; Schmidt *et al.*, 2003 ▸). Intra­molecular distances and angles in these pyrazoles and **1** are very similar and adopt values within a narrow range (Table 1 ▸).

**Figure 2:**
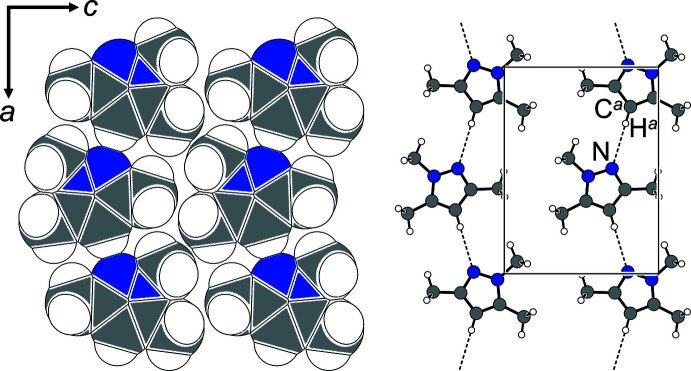
Packing in the (010) plane (Spek, 2020 ▸). Non-classical C—H⋯N contacts are shown as dashed lines: *d*(N⋯H^*a*^) = 2.56 Å; ∠(C^*a*^—H^*a*^⋯N) = 179°. Symmetry code: (*a*) − 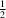 + *x*, 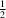 − *y*, 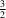 − *z*.

*Pnma*, the space-group type adopted by the title compound, plays a central role in the concepts of Kitaigorodskii and Wilson. We cite literally from Wilson (1991 ▸): ‘The space-group type *Pnma* is particularly inter­esting, as Kitaigorodskii (1965 ▸) predicted that it would be popular because it would permit close-packing of mol­ecules with inherent mirror symmetry […] The structures published in *Acta Crystallographica C* were checked, and all were found to consist of mol­ecules possessing and using inherent mirror planes.’ The 1965 ▸ article cited in Wilson’s statement above refers to the Russian version of *Organic Chemical Crystallography* (Kitaigorodskii, 1961 ▸). The heterocyclic mol­ecule in **1** is a candidate *par excellence* for *Pnma*: It not only matches the required site symmetry but all of its non-hydrogen atoms are located on this mirror plane, providing an efficient in-plane arrangement (Fig. 2 ▸, left).

Non-classical C—H⋯N hydrogen bonds represent the shortest directional contacts in the mirror plane and lead to chains along [100] (Fig. 2 ▸, right). This kind of inter­action is quite common for 4-unsubstituted pyrazoles and we only provide selected examples for comparison: ICEDUQ (Patra *et al.*, 2004 ▸), LUNYID (Benisvy *et al.*, 2009 ▸) and KITNOR (Kidwai *et al.*, 2008 ▸) (Table 2 ▸).

**Figure 3:**
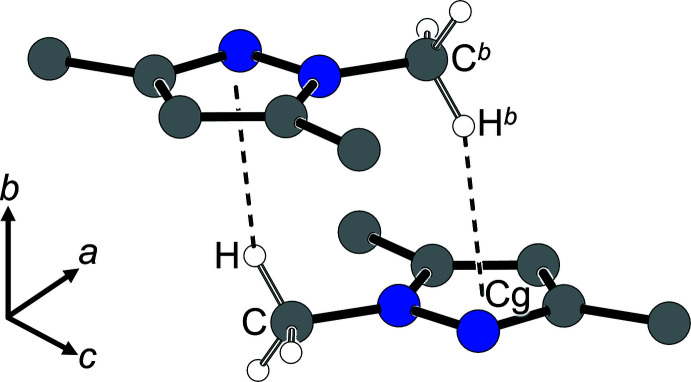
Short methyl C—H⋯π contacts about a center of inversion in **1** shown as dashed lines (Spek, 2020 ▸): *d*(*Cg*⋯H^*b*^) = 2.586 Å; ∠(C^*b*^—H^*b*^⋯*Cg*) = 140.97°. Symmetry code: (*b*) 1 − *x*, −*y*, 1 − *z*.

A crystallographic center of inversion (Wyckoff position 4*a*) relates objects on the mirror planes at *y* = 0.25 and *y* = 0.75; the dipole moments of consecutive layers are therefore oriented in opposite directions, quite in agreement with early Nowacki (1943 ▸) ideas. The non-planar methyl groups in **1** provide the most relevant inter­layer contacts. Fig. 3 ▸ shows the head-to-tail arrangement of two mol­ecules, with a methyl H atom pointing towards the center of gravity of the five-membered ring of a neighbor. The shortest inter­atomic distance associated with this contact amounts to H4*b*⋯N2^*a*^ [symmetry code: (*a*) 1 − *x*, − 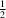 + *y*, 1 − *z*] = 2.65 Å.

**Figure 4:**
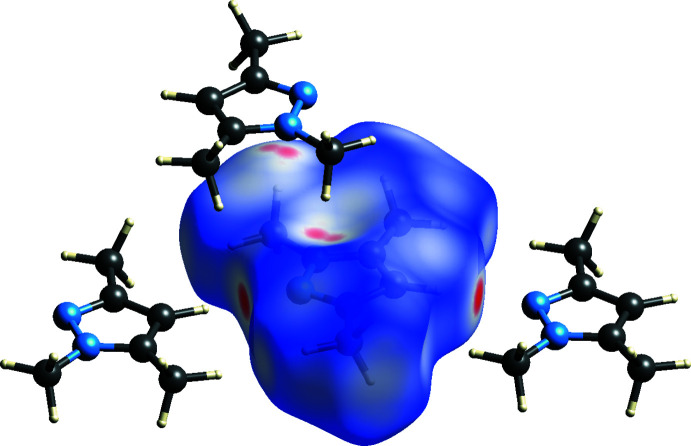
Hirshfeld surface (Turner *et al.*, 2017 ▸) about one 1,3,5-trimethyl-1*H*-pyrazole moiety in **1**.

The Hirshfeld surface (Spackman & Jayatilaka, 2009 ▸) about one pyrazole moiety is shown in Fig. 4 ▸. It has been mapped with the dimensionless inter­action-sensitive qu­antity *d*_norm_; red areas indicate short contacts. Both the C—H⋯N hydrogen bond and the inter­layer meth­yl⋯π contact can clearly be perceived.

**Figure 5:**
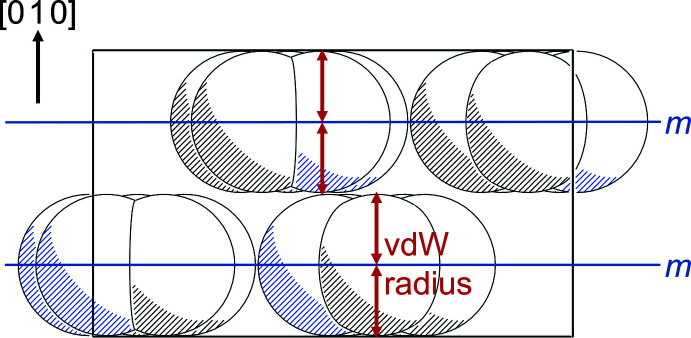
View of the unit cell of **1** along *c* (Spek, 2020 ▸), methyl groups omitted. The radii of the atoms essentially denote their van der Waals radii (*r*_vdW_).

The stacking of efficiently packed layers of which only the methyl H atoms protrude leads to a simple relationship between the lattice parameter in the stacking direction, *i.e.* unit-cell parameter *b* in the standard setting of space group *Pnma*, and the van der Waals radii of the partaking atoms. Fig. 5 ▸ provides a sketch of the situation.

Kitaigorodskii himself had determined van der Waals radii (*r*_vdW_) of 1.8 Å for carbon and of 1.58 Å for nitro­gen (Kitaigorodskii, 1973 ▸); values of 1.7 for C and 1.55 for N have been suggested by Batsanov (1995 ▸). The unit-cell parameter *b* for our title compound **1** amounts to approximately 6.7 Å, closely matching the expected fourfold van der Waals radius of the non-hydrogen atoms involved. Table 3 ▸ shows additional examples for small and planar organic mol­ecules crystallizing in the same space group type and with a similar cell parameter *b*.

These examples share the same construction principle: The individual flat mol­ecules are arranged in the crystallographic mirror plane, and for symmetry reasons dipole directions alternate between consecutive layers along *b*.

## Database survey

For all database searches, version 5.42 of the CSD (Groom *et al.*, 2016 ▸), including all updates until September 2021 were used. The examples compiled in Table 3 ▸ were restricted to entries with space group *Pnma* crystallizing in unit cells similar to **1**, with a tolerance of 0.7 Å for each unit-cell parameter. These conditions were met by seventeen entries; eight of these show a packing analogous to that of **1**.

## Synthesis and crystallization

The target compound 1,3,5-trimethyl-1*H*-pyrazole (**1**) is readily available by the Knorr pyrazole synthesis using acetyl­acetone and methyl­hydrazine (Knorr, 1883 ▸; Stanovnik & Svete, 2002 ▸). Alternatively, the compound may be purchased from common vendors. The single crystal for the reported structure was obtained from the reaction mixture. It is soluble in a wide range of common solvents; single crystals may also be grown *via* recrystallization from a solution in diethyl ether at 243 K. The small crystal size as well as the fast growth and the absence of any heavy atom restricted diffraction data to a limited resolution. The result is a comparatively high agreement factor of symmetry-related reflections (*R*_int_ = 13.77%) and agreement factor considering the intensity of reflections (*R_σ_* = 7.07%).

## Refinement details

Crystal data, data collection parameters and convergence results for the single crystal X-ray diffraction experiment have been summarized in Table 4 ▸. Non-hydrogen atoms were assigned anisotropic displacement parameters. H atoms were introduced into calculated positions and treated as riding with C—H = 0.98 Å and *U*_iso_(H) = 1.5*U*_eq_(C) for methyl and with C—H = 0.95 Å and *U*_iso_(H) = 1.2*U*_eq_(C) for the heteroaryl H atom. Tentative refinement of a model in which the methyl conformations were chosen to best match local difference-Fourier maxima leads to split positions, but for each CH_3_ group one H atom is located very close to the crystallographic mirror plane. We therefore decided to constrain the *y* coordinate of these almost in-plane hydrogens to fit the special position.

## Conclusion and outlook

What else can we learn from the packing of the simple heterocycle **1** in space group *Pnma*. Space filling is unexceptional; according to the well-known Kempster–Lipson rule (Kempster & Lipson, 1972 ▸) a mol­ecule with eight non-hydrogen atoms should be associated with a residue volume of approximately 150 Å^3^. The unit cell of **1** will therefore contain four pyrazole mol­ecules, necessarily in special positions. Wyckoff positions 4*a* and 4*b* require 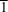 symmetry and can be excluded whereas 4*c* appears compatible with the mol­ecular symmetry. Harker vectors are subtended by atoms related by crystallographic symmetry. All Harker peaks and all Patterson cross peaks (Glusker *et al.*, 1994 ▸; Viterbo, 2002 ▸) derived for occupied 4*c* positions should be characterized by a Patterson coordinate of 0.0 or 0.5 in the [010] direction. The Patterson function for **1** perfectly matches this expectation: The highest Patterson peak with a *v* coordinate unequal to 0.0 or 0.5 has an intensity of less than 5% of the trivial origin peak. Our tri­methyl­pyrazole represents a well-suited example for teaching basic concepts of crystallography such as space groups, Wyckoff positions, packing rules, and popular short contacts!

## Supplementary Material

Crystal structure: contains datablock(s) I, global. DOI: 10.1107/S205698902200860X/dj2050sup1.cif

Structure factors: contains datablock(s) I. DOI: 10.1107/S205698902200860X/dj2050Isup2.hkl

Supporting information file. DOI: 10.1107/S205698902200860X/dj2050Isup3.mol

Suggestion for Table of Contents graphics. DOI: 10.1107/S205698902200860X/dj2050sup4.png

Supporting information file. DOI: 10.1107/S205698902200860X/dj2050Isup5.cml

CCDC reference: 2203895

Additional supporting information:  crystallographic information; 3D view; checkCIF report

## Figures and Tables

**Table 1:** Comparison of selected distances (Å) in **1** with two comparable structures denoted by their CSD refcodes (Groom *et al.*, 2016 ▸) Atom labels as in Fig. 1 ▸. For PYRZOL27, a *Z*′ of 2 is observed and only values for the first residue are listed here.

Compound	*d*(N1—N2)	*d*(N2—C1)	*d*(N1—C3)	*d*(C2—C3)	*d*(C1—C2)
**1**	1.358 (4)	1.351 (4)	1.336 (4)	1.392 (4)	1.360 (5)
PYRZOL27^*a*^	1.357 (2)	1.338 (3)	1.334 (3)	1.391 (3)	1.373 (3)
ALOSE*Z* ^*b*^	1.3464 (17)	1.3595 (14)	1.3415 (16)	1.3966 (15)	1.377 (2)

References: (*a*) Sikora & Katrusiak (2013 ▸); (*b*) Schmidt *et al.* (2003 ▸).

**Table 2:** Comparison of N⋯H—C contacts (Å, °) observed in **1** and selected other pyrazoles

Compound	*d*(N⋯H)	∠(N⋯H—C)
**1**	2.56	179
ICEDUQ^*a*^	2.852 (19)	177.3 (12)
LUNYID^*b*^	2.66	154
KITNOR^*c*^	2.458 (16)	156.2 (13)

References: (*a*) Patra *et al.* (2004 ▸); (*b*) Benisvy *et al.* (2009 ▸); (*c*) Kidwai *et al.* (2008 ▸).

**Table 3:** Other structures showing the same motif as **1**; *r*_vdW_(C) = 1.7 Å; *r*_vdW_(N) = 1.55 Å (Batsanov, 1995 ▸) ^†^For MURANT the non-standard setting *Pbnm* was chosen, so the shown unit-cell parameter perpendicular to the mirror plane is *c*.

Compound	Formula	*b* (Å)	*b*/4 (Å)
**1**	C_6_H_10_N_2_	6.687 (11)	1.672
CIJZEB^*a*^	C_4_H_4_ClN_3_O_2_	6.1372 (5)	1.5343
CIJZEB01^*b*^	C_4_H_4_ClN_3_O_2_	6.3050 (10)	1.5763
EQENU*L* ^*c*^	C_4_H_6_BF_3_N_2_	6.635 (3)	1.659
FIFRAN^*d*^	C_5_H_4_N_2_O_2_	6.388 (2)	1.597
MURANT^*e*†^	C_2_H_7_N_3_O_4_	6.36 (2)	1.59
QOXVII^*f*^	C_7_H_8_N_2_O_2_	6.5670 (7)	1.6418
VORDI*R* ^*g*^	C_4_H_6_N_2_OS	6.4865 (4)	1.6216
WIQLO*X* ^*h*^	C_7_H_8_N_2_O	6.722 (4)	1.681

References: (*a*) Kubicki & Wagner (2007*a* ▸); (*b*) Kubicki & Wagner (2007*b* ▸); (*c*) Takao & Ikeda (2008 ▸); (*d*) Rybalova *et al.* (1998 ▸); (*e*) Bryden (1957 ▸); (*f*) Nawrot *et al.* (2001 ▸); (*g*) Konstanti­nova *et al.* (2014 ▸); (*h*) Aldabbagh *et al.* (1999 ▸).

**Table 4:** Experimental details

Crystal data	
Chemical formula	C_6_H_10_N_2_
*M*_r_	110.16
Crystal system, space group	Orthorhombic, *P* *n* *m* *a*
Temperature (K)	100
*a*, *b*, *c* (Å)	11.205 (19), 6.687 (11), 8.373 (15)
*V* (Å^3^)	627.3 (19)
*Z*	4
Radiation type	Mo *K*α
μ (mm^−1^)	0.07
Crystal size (mm)	0.21 × 0.10 × 0.09

Data collection	
Diffractometer	Bruker *APEX* CCD
Absorption correction	Multi-scan (*SADABS*; Krause *et al.*, 2015 ▸)
*T*_min_, *T*_max_	0.657, 0.745
No. of measured, independent and observed [*I* > 2σ(*I*)] reflections	6665, 648, 366
*R*_int_	0.138

Refinement	
*R*[*F* ^2^ > 2σ(*F* ^2^)], *wR*(*F* ^2^), *S*	0.047, 0.119, 0.87
No. of reflections	648
No. of parameters	50
H-atom treatment	H-atom parameters constrained
Δρ_max_, Δρ_min_ (e Å^−3^)	0.26, −0.23

Computer programs: *SMART* (Bruker, 2001 ▸), *SAINT-Plus* (Bruker, 2009 ▸), *SHELXT2014/5* (Sheldrick, 2015*a* ▸), *SHELXL2019/2* (Sheldrick, 2015*b* ▸), *PLATON* (Spek, 2020 ▸) and *Mercury* (Macrae *at al.*, 2020 ▸).

## supplementary crystallographic information

### Crystal data

**Table:**

C_6_H_10_N_2_	*D*_x_ = 1.166 Mg m^−^^3^
*M**_r_* = 110.16	Mo *K*α radiation, λ = 0.71073 Å
Orthorhombic, *P**n**m**a*	Cell parameters from 263 reflections
*a* = 11.205 (19) Å	θ = 3.0–19.7°
*b* = 6.687 (11) Å	µ = 0.07 mm^−^^1^
*c* = 8.373 (15) Å	*T* = 100 K
*V* = 627.3 (19) Å^3^	Block, colorless
*Z* = 4	0.21 × 0.10 × 0.09 mm
*F*(000) = 240	

### Data collection

**Table:**

Bruker APEX CCD diffractometer	648 independent reflections
Radiation source: microsource	366 reflections with *I* > 2σ(*I*)
Multilayer optics monochromator	*R*_int_ = 0.138
ω scans	θ_max_ = 25.9°, θ_min_ = 3.0°
Absorption correction: multi-scan (SADABS; Krause *et al.*, 2015)	*h* = −13→13
*T*_min_ = 0.657, *T*_max_ = 0.745	*k* = −8→8
6665 measured reflections	*l* = −10→10

### Refinement

**Table:**

Refinement on *F*^2^	Hydrogen site location: mixed
Least-squares matrix: full	H-atom parameters constrained
*R*[*F*^2^ > 2σ(*F*^2^)] = 0.047	*w* = 1/[σ^2^(*F*_o_^2^) + (0.0645*P*)^2^] where *P* = (*F*_o_^2^ + 2*F*_c_^2^)/3
*wR*(*F*^2^) = 0.119	(Δ/σ)_max_ < 0.001
*S* = 0.87	Δρ_max_ = 0.26 e Å^−^^3^
648 reflections	Δρ_min_ = −0.23 e Å^−^^3^
50 parameters	Extinction correction: SHELXL-2019/2 (Sheldrick 2015b), Fc^*^=kFc[1+0.001xFc^2^λ^3^/sin(2θ)]^-1/4^
0 restraints	Extinction coefficient: 0.011 (4)
Primary atom site location: dual	

### Special details

**Table:**

Geometry. All esds (except the esd in the dihedral angle between two l.s. planes) are estimated using the full covariance matrix. The cell esds are taken into account individually in the estimation of esds in distances, angles and torsion angles; correlations between esds in cell parameters are only used when they are defined by crystal symmetry. An approximate (isotropic) treatment of cell esds is used for estimating esds involving l.s. planes.

### Fractional atomic coordinates and isotropic or equivalent isotropic displacement parameters (Å^2^)

**Table:**

	*x*	*y*	*z*	*U*_iso_*/*U*_eq_	
N1	0.4912 (2)	0.250000	0.6988 (3)	0.0289 (7)	
N2	0.5279 (2)	0.250000	0.5442 (3)	0.0249 (6)	
C1	0.6481 (3)	0.250000	0.5301 (4)	0.0236 (7)	
C2	0.6912 (3)	0.250000	0.6820 (3)	0.0260 (8)	
H2	0.772711	0.250000	0.713077	0.031*	
C3	0.5924 (3)	0.250000	0.7829 (4)	0.0265 (7)	
C4	0.4409 (3)	0.250000	0.4164 (3)	0.0327 (9)	
H4A	0.360267	0.250000	0.461847	0.049*	
H4B	0.451701	0.130335	0.350369	0.049*	
C5	0.7079 (3)	0.250000	0.3728 (4)	0.0342 (8)	
H5A	0.794608	0.250000	0.388049	0.051*	
H5B	0.684366	0.369665	0.312985	0.051*	
C6	0.5878 (3)	0.250000	0.9617 (4)	0.0380 (9)	
H6A	0.504403	0.250000	0.996905	0.057*	
H6B	0.628021	0.369665	1.002625	0.057*	

### Atomic displacement parameters (Å^2^)

**Table:**

	*U*^11^	*U*^22^	*U*^33^	*U*^12^	*U*^13^	*U*^23^
N1	0.0378 (16)	0.0195 (13)	0.0295 (16)	0.000	0.0012 (14)	0.000
N2	0.0297 (15)	0.0182 (13)	0.0268 (15)	0.000	−0.0012 (12)	0.000
C1	0.0238 (16)	0.0149 (15)	0.032 (2)	0.000	0.0009 (14)	0.000
C2	0.0263 (17)	0.0177 (15)	0.034 (2)	0.000	−0.0025 (16)	0.000
C3	0.0356 (17)	0.0166 (14)	0.0274 (18)	0.000	−0.0035 (17)	0.000
C4	0.038 (2)	0.0228 (15)	0.037 (2)	0.000	−0.0104 (16)	0.000
C5	0.040 (2)	0.0275 (16)	0.035 (2)	0.000	0.0047 (16)	0.000
C6	0.048 (2)	0.0349 (17)	0.031 (2)	0.000	0.0033 (17)	0.000

### Geometric parameters (Å, º)

**Table:**

N1—C3	1.336 (4)	C4—H4A	0.9800
N1—N2	1.358 (4)	C4—H4B	0.9800
N2—C1	1.351 (4)	C4—H4B^i^	0.9800
N2—C4	1.448 (4)	C5—H5A	0.9800
C1—C2	1.360 (5)	C5—H5B	0.9800
C1—C5	1.478 (4)	C5—H5B^i^	0.9800
C2—C3	1.392 (4)	C6—H6A	0.9800
C2—H2	0.9500	C6—H6B	0.9800
C3—C6	1.498 (5)	C6—H6B^i^	0.9800
			
C3—N1—N2	104.2 (3)	N2—C4—H4B^i^	109.47 (10)
C1—N2—N1	112.7 (2)	H4A—C4—H4B^i^	109.5
C1—N2—C4	127.3 (3)	H4B—C4—H4B^i^	109.5
N1—N2—C4	120.0 (3)	C1—C5—H5A	109.5
N2—C1—C2	105.8 (3)	C1—C5—H5B	109.5
N2—C1—C5	122.0 (3)	H5A—C5—H5B	109.5
C2—C1—C5	132.2 (3)	C1—C5—H5B^i^	109.47 (11)
C1—C2—C3	106.6 (3)	H5A—C5—H5B^i^	109.5
C1—C2—H2	126.7	H5B—C5—H5B^i^	109.5
C3—C2—H2	126.7	C3—C6—H6A	109.5
N1—C3—C2	110.8 (3)	C3—C6—H6B	109.5
N1—C3—C6	119.9 (3)	H6A—C6—H6B	109.5
C2—C3—C6	129.4 (3)	C3—C6—H6B^i^	109.47 (10)
N2—C4—H4A	109.5	H6A—C6—H6B^i^	109.5
N2—C4—H4B	109.5	H6B—C6—H6B^i^	109.5
H4A—C4—H4B	109.5		

Symmetry code: (i) *x*, −*y*+1/2, *z*.

### Comparison of selected distances in 1 with two comparable structures.

For PYRZOL27 a *Z*' of 2 is observed and only values for the first residue have been taken into account.

**Table:**

Compound	*d*(N—N)	*d*1(N═C)	*d*2(N═C)	*d*1(C═C)	*d*2(CC)
1	1.358 (4)	1.351 (4)	1.336 (4)	1.392 (4)	1.360 (5)
PYRZOL27	1.357 (2)	1.338 (3)	1.334 (3)	1.391 (3)	1.373 (3)
ALOSEZ	1.3464 (17)	1.3595 (14)	1.3415 (16)	1.3966 (15)	1.377 (2)
