# Supplementary figures and images for: Tri­methyl­pyrazole: a simple heterocycle reflecting Kitaigorodskii’s packing principle
Source: Acta Crystallogr E Crystallogr Commun. 2022 Sep 2;78(Pt 10):966–70. doi: 10.1107/S205698902200860X (PMC9535818; doi:10.1107/S205698902200860X)

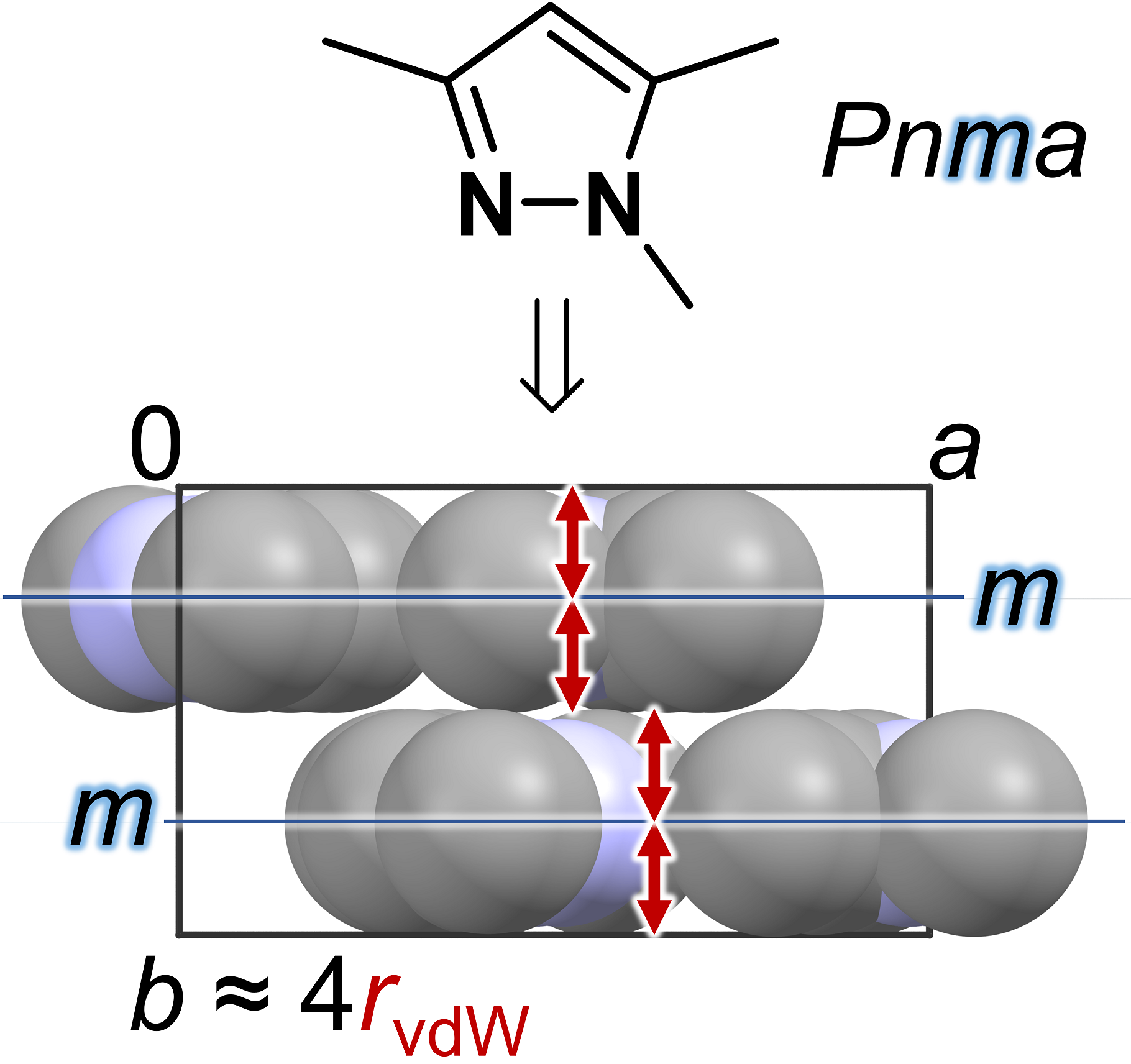

Supplement: Supplementary file 4 [file e-78-00966-sup4.png]
